# Storage and persistence of a candidate fungal biopesticide for use against adult malaria vectors

**DOI:** 10.1186/1475-2875-11-354

**Published:** 2012-10-25

**Authors:** Simon Blanford, Nina E Jenkins, Riann Christian, Brian HK Chan, Luisa Nardini, Michael Osae, Lizette Koekemoer, Maureen Coetzee, Andrew F Read, Matthew B Thomas

**Affiliations:** 1 Center for Infectious Disease Dynamics, Penn State University, Department of Biology, Mueller Laboratory, University Park, PA, 16802, USA; 2 Center for Infectious Disease Dynamics, Department of Entomology, Penn State University, Merkle Lab, University Park, PA, 16802, USA; 3 Vector Control Reference Unit, National Institute for Communicable Diseases of the NHLS, Private Bag X4, Sandringham, Johannesburg, 2131, South Africa; 4 Malaria Entomology Research Unit, School of Pathology, Faculty of Health Sciences, University of Witwatersrand, Johannesburg, South Africa; 5 Fogarty International Center, National Institutes of Health, Bethesda, MD, 20892, USA

## Abstract

**Background:**

New products aimed at augmenting or replacing chemical insecticides must have operational profiles that include both high efficacy in reducing vector numbers and/or blocking parasite transmission and be long lasting following application. Research aimed at developing fungal spores as a biopesticide for vector control have shown considerable potential yet have not been directly assessed for their viability after long-term storage or following application in the field.

**Methods:**

Spores from a single production run of the entomopathogenic fungi *Beauveria bassiana* were dried and then stored under refrigeration at 7°C. After 585 days these spores were sub-sampled and placed at either 22°C, 26°C or 32°C still sealed in packaging (closed storage) or in open beakers and exposed to the 80% relative humidity of the incubator they were kept in. Samples were subsequently taken from these treatments over a further 165 days to assess viability. Spores from the same production run were also used to test their persistence following application to three different substrates, clay, cement and wood, using a hand held sprayer. The experiments were conducted at two different institutes with one using adult female *Anopheles stephensi* and the other adult female *Anopheles gambiae*. Mosquitoes were exposed to the treated substrates for one hour before being removed and their survival monitored for the next 14 days. Assays were performed at monthly intervals over a maximum seven months.

**Results:**

Spore storage under refrigeration resulted in no loss of spore viability over more than two years. Spore viability of those samples kept under open and closed storage was highly dependent on the incubation temperature with higher temperatures decreasing viability more rapidly than cooler temperatures. Mosquito survival following exposure was dependent on substrate type. Spore persistence on the clay substrate was greatest achieving 80% population reduction for four months against *An. stephensi* and for at least five months against *Anopheles gambiae*. Cement and wood substrates had more variable mortality with the highest spore persistence being two to three months for the two substrates respectively.

**Conclusions:**

Spore shelf-life under refrigeration surpassed the standard two year shelf-life expected of a mosquito control product. Removal to a variety of temperatures under either closed or open storage indicated that samples sent out from refrigeration should be deployed rapidly in control operations to avoid loss of viability. Spore persistence following application onto clay surfaces was comparable to a number of chemical insecticides in common use. Persistence on cement and wood was shorter but in one assay still comparable to some organophosphate and pyrethroid insecticides. Optimized formulations could be expected to improve spore persistence still further.

## Background

The increasing incidence of resistance to chemical insecticides by malaria mosquito vectors [1-5] has prompted a wide-ranging search for alternative control tools. This research includes various projects investigating the control potential of a range of insect pathogens and parasites [6-10]. Among these novel biocontrol approaches, the use of entomopathogenic fungi formulated as biopesticides has received considerable attention [11-20]. Numerous studies show that fungi can infect adult mosquitoes when applied to a range of substrates, suggesting potential for use as Indoor Residual Sprays (IRS) [6,7,20], or via novel delivery strategies such as impregnated eve curtains [19], resting targets [11,21] or even impregnated bed nets [20,22]. While slower acting than conventional chemical insecticides, fungal infection has been shown to cause substantial reduction in transmission potential of a range of vector species [6,7,23-26]. Further, insecticide resistant mosquitoes appear to remain fully susceptible to fungal infection [7,17,18].

To date, most studies exploring fungal infection in adult mosquitoes have focused on direct measures of efficacy (e.g. mosquito mortality, biting rate, fecundity etc.) and on the whole, the data are encouraging. However, ultimate development of a practical control tool requires understanding of several performance metrics and not just efficacy. Key among these is product stability, including the storage potential of spores (the active ingredient) post-production and the persistence of spores following application.

The World Health Organization stipulates that chemical insecticides used for mosquito vector control should be able to be stored for two years without significant loss in efficacy of the active ingredient [27,28]. Previous studies on entomopathogenic fungi suggest that the strain, the production conditions, temperature, humidity and spore moisture content can all influence fungal viability during long-term storage [29-34]. Under ideal conditions of low temperature (*c*.5°C) and low spore moisture content (<5%), entomopathogenic fungi have been shown to store with minimal loss of viability for over 2 years, as long as initial viability is high [30]. Such longevity looks promising but determining the storage potential of particular candidate strains is an important step in evaluating operational potential of the mosquito biopesticide approach.

With respect to persistence, how long a product needs to remain viable after treatment will likely depend on the delivery system. Intuitively, the longer a product lasts the better but there are no precedents for novel delivery strategies such as point source applications on a small resting target [19,21] and in principle, a product could be re-applied relatively frequently without major disruption to behavior or substantial cost. For established approaches such as IRS, on the other hand, there are targets set by WHO based on existing product profiles. At present, the current insecticides approved for use in IRS by WHO persist for 2–6 months after application, with DDT setting the ‘gold standard’ [35-37].

Studies investigating persistence of fungal spores have largely focused on decay rates of biopesticides applied in agricultural settings. In many cases infective half-lives are on the order of days or weeks [38-40]. The chief reason for this rapid decay is that fungal spores are very quickly deactivated by solar (UV) radiation [41]. When spores are applied in more protected environments, such as soil inoculation, persistence can be extended to months or even years [42,43]. Since the application of fungal biopesticides for mosquito control will most likely be in indoor (or at least shaded) environments, UV radiation is unlikely to be a constraint suggesting that persistence might good. Again, however, it is necessary to determine this for any candidate biopesticide.

The aim of the current study is to examine the storage and persistence of an isolate of *Beauveria bassiana* that is one of the leading candidates for development as a mosquito biopesticide product. The viability of spores maintained under a range of conditions in the lab was quantified to determine storage potential at different points along a pesticide ‘supply chain’. Spores retained high viability for over two years under good storage conditions. Transfer to less ideal conditions after this period resulted in decline in viability over a period of one to three months depending on temperature. Product persistence on a range of substrates simulating an IRS-type application was examined by conducting one set of assays with *An. stephensi* and a second set with *An. gambiae*. Fungal spray residues remained infective on clay substrates for 4–6 months, performing much better than a pyrethroid toxic standard. Performance could be extended if mosquitoes received more than one exposure. Persistence on wood and concrete was less good, and varied from as little as 1 week up to 2 or 3 months depending on the particular assay. The results are discussed in the context of further research and development priorities to progress the fungal biopesticide approach towards operational use. There is considerable scope to increase persistence with appropriate formulation chemistry.

## Methods

### Fungal maintenance, production and storage methods and tests

*Beauveria bassiana* isolate I93-825 was maintained in long-term storage at −80°C on microporous beads (Pro-Lab Diagnostics, Austin, Texas, USA). Prior to use, the fungus was recovered by placing one or two beads onto Potato dextrose agar (Oxoid, UK) or Sabouraud dextrose agar (Oxoid, UK) in 9 cm diameter Petri dishes or slopes in 25 ml Universal bottles and incubated at 25°C for 10 days.

### Mass production

Conidia were harvested from slopes or plates to make a spore suspension of approximately 1 x 10^6^ spores ml^–1^ in sterile 0.05% w/v Tween 80 (Sigma) in distilled water. One ml of this suspension was then used to inoculate 75 ml sterile liquid culture medium (4% d-Glucose, 2% yeast extract [Oxoid, UK] in tap water), in 250 ml Erlenmyer flasks. Flasks were incubated on a rotary shaker (200 rpm) at 22°C for 3 days.

Organic barley flakes were weighed into mushroom spawn bags (Unicorn, Garland, Texas, USA) with 1 kg per bag. 600 ml of tap water was then added to each bag and the contents mixed by hand to ensure even absorption of the water. The spawn bags were then placed inside autoclave bags for protection and autoclaved for 30 min at 121°C. Once cool the bags were inoculated under aseptic conditions with 75 ml of the 3-day-old liquid medium (above) plus 75 ml of sterile water to achieve a final moisture content of approximately 48%. The inoculated bags were carefully massaged to ensure even distribution of the inoculum. The bags were then sealed and incubated on shelves for 10 days at 22°C. Following incubation, the bags were opened in a reverse flow cabinet (Labconco, USA) and the contents transferred to brown paper bags for drying. The paper bags were placed in a dehumidified room for 4 days (22°C), until the sporulated substrate reached <20% moisture content. The spores were then harvested from the barley flakes using a Mycoharvester (Acis Manufacturing, Devon, UK). The harvested spores were placed in glass dishes and further dried in a desiccator over dry silica gel at 22°C. Once the spore powder reached 5% moisture content, a small sample was taken for quality analysis and the remaining powder was sealed in foil laminated sachets (P39 moisture barrier film) and stored at 7°C. The following storage and persistence studies used spores from a large production run that had been in storage for 585 days prior to the onset of testing. At this point spores were 98% viable (see germination test methods below).

### Long-term storage

Dried spores were taken from storage and distributed into small (5 x 5 cm) hermetically sealed foil laminate sachets. A subset was placed back under the original storage conditions and their germination measured at the end of the study period (day 750).

Other sachets were placed in three environmental chambers maintained at 22°C, 26°C or 32°C to simulate transfer of spores from long-term storage into labs or warehouses for distribution or formulation. Individual sachets were removed at approximately weekly intervals and destructively sampled for germination.

To investigate the interaction between temperature and humidity, other spores were removed from storage and placed in open 200 ml glass beakers (10 g dry spore powder per beaker) and then transferred to the environmental chambers set at 22, 26 and 32°C. Because the beakers were open, these spores were exposed to ambient relative humidity in the chambers, which was set at 80% to represent typical tropical conditions. Viability was checked over time by taking sub-samples of spore powder from the beakers at approximately weekly intervals to test germination rate.

### Germination tests

To assess viability of spores, a small sample of spore powder was suspended in Isopar M (ExxonMobil) to a concentration of approximately 1 x 10^7^ sp ml^-1^. One drop of this suspension was transferred onto SDA in 6 cm diameter Petri plates using a microspatular and spread evenly over the surface of the agar. Three replicates were prepared for each sample time and incubated at 25°C for 20 hr. After incubation, the spores on the agar surface were examined under a compound microscope (at 500x magnification). Spores were counted as germinated if a germ was visibly protruding from the spore. All spores in each field of view were assessed. A total of at least 300 spores were counted per plate and viability was calculated as a percentage of the total.

### Fungal persistence after application

#### Basic assay methods

The World Health Organization Pesticide Evaluation Scheme (WHOPES) prescribes a set of standardized protocols for evaluating persistence of compounds for use in IRS [35]. Each compound must be applied to three different substrates (clay/mud, cement/concrete and wood) and mosquitoes exposed to the treated surfaces for one hour (depending on species) at different time points after application. For conventional fast acting chemical pesticides, the number of insects knocked down after the exposure period and then the number dead after 24 hours are recorded. The WHO cut-off point for acceptable mortality is 80%. This general protocol was followed but with one important modification to account for the very different mode of action of fungal entomopathogens that cause mortality over a timeframe of 1–2 weeks rather than 24h (note, that it takes about 14 days for the malaria parasite to develop within the mosquito, so that products with slower mortality can still provide excellent malaria control by shortening mosquito life span to prevent successful parasite incubation [6,7,24-26]. In line with earlier studies [6,7] we monitored mortality for 14 days, with persistence assessments discontinued when a product failed to achieve the 80% mortality endpoint. Persistence was assessed under standard WHOPES conditions of 26 ± 2°C, 80 ± 10% RH and 12:12 day:night cycle.

#### Test substrates

Blocks of cement (Quickrete®) and clay (White Earthenware; Clay-King Inc.), each 5 mm thick, were prepared in 15cm Petri dishes and dried at 25 ± 2°C and 50 ± 10% RH for at least 6 weeks prior to testing. A third substrate, wood tiles, 5 mm thick 16 x 16 cm square blocks (birch plywood; Lowes Inc.), was also included. Following application all substrates were stored unsealed and therefore open to the test temperature and humidity (see above) for the duration of their use in each experiment.

### Mosquito exposure to the substrates

A standard WHO cone assay [35] was used for exposing the mosquitoes to the substrates. The plastic cone was secured over each tile and approximately 30 unfed 3–5 day old female mosquitoes introduced. The mosquitoes were left for one hour and then subsequently removed to holding cups (0.33 liter cardboard drinking cups with mosquito mesh cover for the lids) where they were maintained on 10% glucose water. Each treatment for all assays was replicated four times giving a minimum of 120 mosquitoes per assay. Mortality was monitored daily and dead insects removed from the cages.

#### Persistence assay 1 – *Anopheles stephensi*

The first persistence assay was conducted at Penn State using a long-standing laboratory colony of *An. stephensi*. Spores of *B. bassiana* were suspended in a mix of mineral oils (80% Isopar M: 20% Ondina 22) and the concentration adjusted to give our standard lab dose of 1x10^9^ spores/ml. Fungal spores are lipophylic and this mix of oils acts as a simple carrier with appropriate viscosity for spraying. Spores were applied using a small hand-held pump sprayer clamped horizontally 10cm above the test tile. The pump sprayer comprised a pump spray cap (Calmar Inc.) screwed onto a 25 ml glass universal vial. Each tile received five pumps from the sprayer, which delivered a total of 3.5 ml of formulation to give an equivalent dose rate of 3.3x10^11^ spores/m^2^.

As a positive control we used the pyrethroid insecticide Lambda-cyhalothrin (Chem Service Inc.) at a dose rate of 30mg/ml a.i. Acetone acted as the solvent for the insecticide and silicon oil (Dow Corning 554) served as the carrier. We used technical a.i. in an oil carrier rather than a proprietary formulation, such as a capsulated suspension, because the fungus itself is applied in a simple carrier oil and there are, as yet, no more advanced formulations for comparison. The concentrations were calculated based on the mg of active ingredient per unit of oil [35]. The insecticide was applied in the same manner as the biopesticide to each of the three substrates. Negative controls comprised untreated tiles.

Exposures were performed on Day 1 (i.e. allowing 24 hrs for the treated surfaces to dry) and then every month until the biopesticide failed to hit the 80% mortality target. In addition, at five months post spray (when the fungal spray residue had decayed considerably – see results), a repeat exposure experiment was conducted using the clay substrate to simulate what might happen if mosquitoes contacted a treated surface across successive feeding cycles, rather than just once. To do this an extra treatment group was added and exposed to the same treated clay substrate as was used for other exposures. This group was exposed on day 0, immediately after the single exposure Month 5 group were exposed, and then again three days later, again six days after the initial exposure and finally nine days after the initial exposure. At all other times this group was housed and maintained as all other treatment groups described above.

#### Persistence assay 2 – *Anopheles gambiae*

A repeat persistence study was conducted using *An. gambiae* (insecticide susceptible SUA strain) at the Vector Control Reference Unit, National Institute for Communicable Diseases, South Africa. The study used the same fungus and substrates and followed the same protocols as described above but because of demands on the insect colony, did not include a pyrethroid as a positive control.

### Statistical analysis

Spore survival during storage was summarized as mean percent germination from the three replicates counted at each time point. A Weibull survival function was fitted to the data for spores incubated at different temperatures for both closed and open storage treatments:

$$S=\exp\left[-\left(A/G\right)t^{G}\right]$$

where S is proportional spore germination, A and G are best-fit parameters and *t* is time. These curves were used to estimate the LT_20_ (i.e. when germination was reduced to 80%, the accepted minimum viability for operational use [44]) and the LT_50_ (time to 50 percent germination).

Mosquito survival data were analysed using a Kaplan-Meier survival analysis in SPSS for Mac (v.19) with significant differences between doses and/or treatments estimated using a Log Rank Test.

## Results

### Spore viability during storage

**Figure 1:**
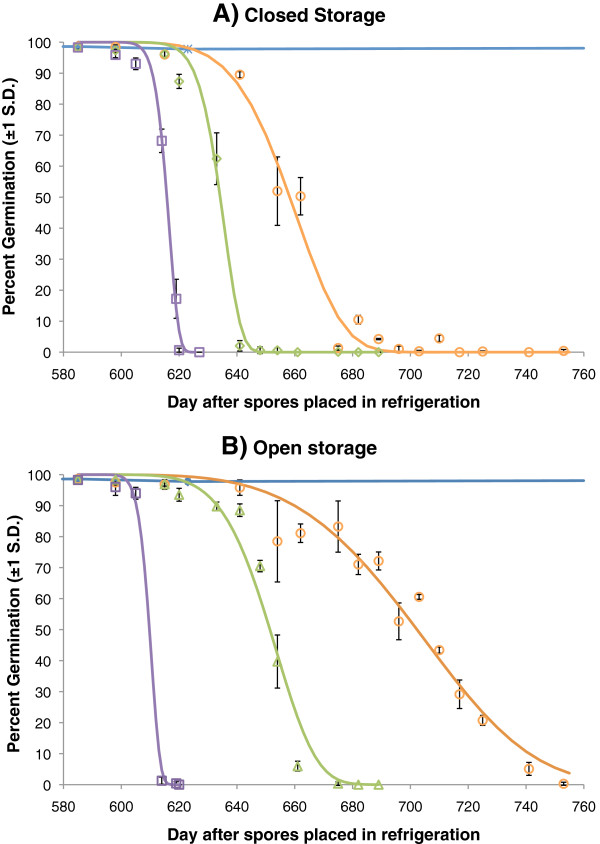
**Germination (percent) of spores of *Beauveria bassiana *after long term storage under varying conditions. A**) Germination of spores removed from 7°C after 580 days and subsequently maintained at 22°C, 26°C or 32°C. **B**) As for **A**) but spores were taken from the refrigeration conditions after 580 days and subsequently maintained in open glass beakers placed in incubators with relative humidity at 80% and temperatures of 22°C, 26°C and 32°C. Blue line is for germination of spores at 7°C across the monitoring period (same for both graphs as a reference), orange points spores kept at 22°C, green points spores kept at 26°C and purple points spores kept at 32°C. Curves are Weibull survival functions fitted through the germination data at each temperature. *R*^*2*^ and parameter values can be found in Table 1.

Spores of *Beauveria bassiana* dried to 5% moisture content and maintained in sealed aluminium sachets at 7°C showed no loss in viability over the monitoring period, with spore germination rate of 98.1 ±0.55% after 750 days (Figure 1A, B).

Spores taken from refrigeration and placed at different temperatures showed a clear temperature dependent decline in viability (Figure 1A), with decay half-lives of 31, 49 and 71 days for 32°C, 26°C and 22°C, respectively (Table 1).

**Table 1:** Model parameters and survival data for spore viability when kept in sealed or open storage at different temperatures

**Treatment**	**Storage temperature**	**Weibull parameters**			**Spore survival (days)**	
		**A**	**G**	***R***^***2***^	**LT**_**20**_	**LT**_**50**_
Closed storage	22°C	1.53x10^-10^	5.578	0.99	60	73
	26°C	6.05x10^-16^	9.477	0.99	44	49
	32°C	1.78x10^-15^	10.49	0.99	28	31
Open Storage	22°C	5.85x10^-9^	4.203	0.96	90	117
	26°C	7.76x10^-11^	5.883	0.98	55	67
	32°C	5.94x10^-13^	9.357	0.99	22	25

Spores were moved from refrigeration after 585 days and sub-sampled at intervals until germination rates had declined to zero. Details of the Weibull survival function are given in the main text.

Removal from refrigeration to warm temperatures and high ambient humidity also resulted in decline in viability (Figure 1B), although decay rates were relatively slower for the cooler temperatures than under sealed conditions (LT_50s_ of 67 and 117 days for 26°C and 22°C, respectively (Table 1)).

### Fungal persistence following application

#### Assay 1 – *An. stephensi*

**Figure 2:**
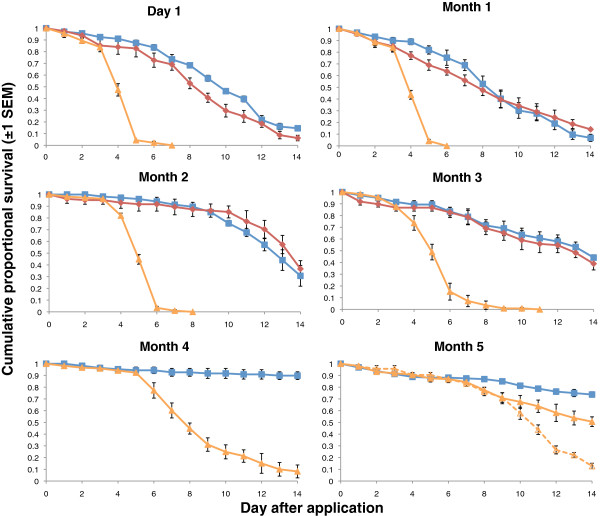
**Cumulative proportional survival of *Anopheles stephensi *exposed to spores of *B. bassiana *or the pyrethroid insecticide lambda-cyhalothrin applied to clay tiles.** Control tiles were left untreated. The chemical pesticide was dropped from assessments after month 3 as it was not achieving the specified mortality level. Exposures were carried out monthly after an initial post application assessment (Day 1) for five months. Blue, red and orange curves show survival for the control, pyrethroid and *B. bassiana* treatments respectively. The dotted orange curve in the Month 5 figure shows survival of *An. stephensi* which were exposed every three days to the *B. bassiana* treated clay tiles (see main text for further details).

Spray applications of *B. bassiana* achieved 80% mortality for four months on the clay substrate (Table 2 and Figure 2). At month five *An. stephensi* survival was still significantly less than control survival but did not decline below 20% by the end of the 14 day assessment period. Repeated exposure of mosquitoes on a three day ‘feeding cycle’, however, restored efficacy and resulted in 80% by day 13 (Table 2 and Figure 2). In contrast the pyrethroid Lambda-cyhalothrin performed very poorly on clay, with no obvious knockdown and very little difference in overall survival to the control group. The insecticide was dropped from assessments after month 3.

**Table 2:** ***Anopheles stephensi *survival after exposure to clay tiles**

**Time after exposure**	**Treatment**	Median Lethal Time days (95% C.I.)*	**Log rank statistic (Significance compared to control)**	Time to 80% mortality days (± 1 SEM)*
**Day 1**	**Control**	10.0 (9.05-10.95)	---------	12.8 (± 0.48)
	**Pyrethroid**	9.0 (8.25-9.75)	5.9 (*P* = 0.015)	12.3 (± 0.48)
	***B. bassiana***	4.0 (3.74-4.26)	164.1 (*P* < 0.001)	5.0 (± 0.00)
**Week 1**	**Control**	13.0 (11.92-14.01)	---------	Not achieved
	**Pyrethroid**	13.0 (11.0-15.0)	0.63 (*P* = 0.43)	Not achieved
	***B. bassiana***	4.0 (3.73-4.27)	107.5 (*P* < 0.001)	5.3 (± 0.25)
**Month 1**	**Control**	13.0 (12.23-13.77)	---------	12.3 (± 0.25)
	**Pyrethroid**	14.0 (13.52-14.48)	2.05 (*P* = 0.15)	12.5 (± 0.96)
	***B. bassiana***	5.0 (4.77-5.23)	190.2 (*P* < 0.001)	5.0 (± 0.00)
**Month 2**	**Control**	14.0 (12.88-15.12)	---------	Not achieved
	**Pyrethroid**	13.0 (11.20-14.80)	0.85 (*P* = 0.36)	Not achieved
	***B. bassiana***	5.0 (4.68-5.32)	162.37 (*P* < 0.001)	6.0 (± 0.00)
**Month 3**	**Control**	10.0 (9.07-10.93)	---------	Not achieved
	**Pyrethroid**	9.0 (8.25-9.75)	5.7 (*P* = 0.017)	Not achieved
	***B. bassiana***	4.0 (3.74-4.26)	168.7 (*P* < 0.001)	6.5 (± 0.50)
**Month 4**	**Control**	Not achieved	---------	Not achieved
	***B. bassiana***	9.0 (8.40-9.60)	144.5 (*P* < 0.001)	12.3 (± 1.03)
**Month 5**	**Control**	Not achieved	---------	Not achieved
	***B. bassiana***	Not achieved	18.7 (*P* < 0.001)	Not achieved
	***B. bassiana *(repeat exposure)**	11.0 (10.42-11.58)	119.8 (*P* < 0.001)	13.3 (± 0.48)

‘Not achieved’ indicates that the desired level of mortality (Median Lethal Time or 80%) was not reached before the end of the 14 day monitoring period.

**Figure 3:**
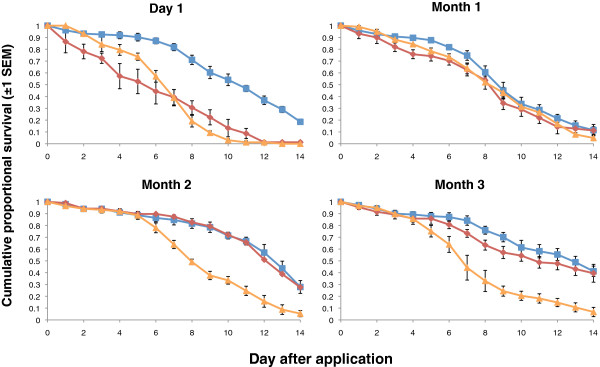
**Cumulative proportional survival of *Anopheles stephensi *exposed to spores of *B. bassiana *or the pyrethroid insecticide Lambda-cyhalothrin applied to cement tiles.** Control tiles were left untreated. The chemical pesticide was dropped from assessments after month 3 as it was not achieving the specified mortality level. Exposures were carried out monthly after an initial post application assessment (Day 1) for five months. Blue, red and orange curves show survival for the control, pyrethroid and *B. bassiana* treatments respectively.

On cement, 80% mortality was achieved for three months, though mosquitoes were clearly infected up to month 5 as indicated by survival differences relative to controls (Table 3 and Figure 3). Similar to the clay substrate, after the initial assessment on day 1, the pyrethroid performed poorly on cement and was dropped from assessments after 3 months.

**Table 3:** ***Anopheles stephensi *survival after exposure to cement tiles**

**Time after exposure**	**Treatment**	Median Lethal Time days (95% C.I.)*	**Log rank statistic (Significance compared to control)**	Time to 80% mortality days (± 1 SEM)*
**Day 1**	**Control**	11.0 (9.89-12.11)	---------	Not achieved
	**Pyrethroid**	6.0 (4.77-7.23)	58.5 (*P* < 0.001)	10 (± 0.82)
	***B. bassiana***	7.0 (6.45-7.55)	89.1 (*P* < 0.001)	11.0 (± 0.71)
**Week 1**	**Control**	12.0 (10.55-13.45)		Not achieved
	**Pyrethroid**	Not achieved	4.1 (P = 0.042)	Not achieved
	***B. bassiana***	7.0 (5.99-8.02)	42.2 (P < 0.001)	Not achieved
**Month 1**	**Control**	9.0 (8.25-9.75)	---------	12.8 (± 0.63)
	**Pyrethroid**	9.0 (8.27-9.73)	0.96 (*P* = 0.33)	12.0 (± 0.71)
	***B. bassiana***	9.0 (8.07-9.93)	2.48 (*P* = 0.12)	12.0 (± 0.41)
**Month 2**	**Control**	13.0 (12.36-13.64)	---------	Not achieved
	**Pyrethroid**	13.0 (12.22-13.78)	0.01 (*P* = 0.92)	Not achieved
	***B. bassiana***	8.0 (7.33-8.67)	52.63 (*P* < 0.001)	12.0 (± 0.58)
**Month 3**	**Control**	13.0 (11.52-14.84)	---------	Not achieved
	**Pyrethroid**	11.0 (8.72-13.28)	1.8 (*P* = 0.18)	Not achieved
	***B. bassiana***	7.0 (6.55-7.45)	63.3 (*P* < 0.001)	11.0 (± 1.29)
**Month 4**	**Control**	Not achieved		Not achieved
	**Pyrethroid**	---------	---------	---------
	***B. bassiana***	Not achieved	16.5 (*P* < 0.001)	Not achieved
**Month 5**	**Control**	Not achieved	---------	Not achieved
	**Pyrethroid**	---------	---------	Not achieved
	***B. bassiana***	Not achieved	4.4 (*P* = 0.037)	Not achieved

‘Not achieved’ indicates that the desired level of mortality (Median Lethal Time or 80%) was not reached before the end of the 14 day monitoring period.

**Figure 4:**
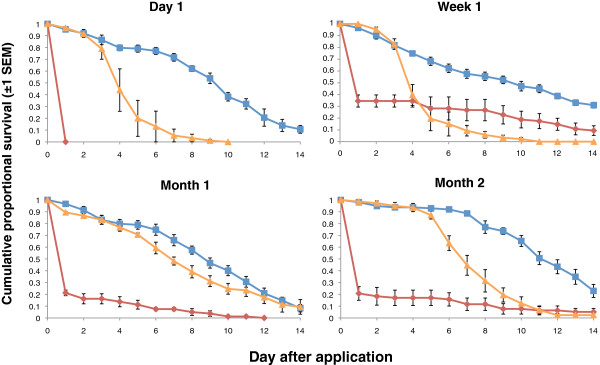
**Cumulative proportional survival of *Anopheles stephensi *exposed to spores of *B. bassiana *or the pyrethroid insecticide Lambda-cyhalothrin applied to wood tiles.** Control tiles were left untreated. Exposures were carried out monthly after an initial post application assessment (Day 1) for five months. Blue, red and orange curves show survival for the control, pyrethroid and *B. bassiana* treatments respectively.

On wood, 80% mortality was achieved for 2 months with evidence of infection up to month 4 (Table 4 and Figure 4). In contrast to the other two substrates the pyrethroid generally produced rapid mortality in the mosquitoes twenty-four hours after exposure. The absolute level of mortality achieved was variable with 100% mortality achieved within 24 hours of exposure on day 1 but only 22% mortality in month 4.

**Table 4:** ***Anopheles stephensi *survival after exposure to wood tiles**

**Time after exposure**	**Treatment**	**Median Lethal Time in days (95% C.I.)**	**Log rank statistic (Significance compared to control)**	**Time to 80% mortality days (± 1 SEM)**
**Day 1**	**Control**	10.0 (9.34-10.66)	---------	12.8 (± 0.48)
	**Pyrethroid**	1.0 (0.00-0.00)	198.7 (*P* < 0.001)	1.0 (± 0.00)
	***B. bassiana***	4.0 (3.64-4.37)	105.7 (*P* < 0.001)	5.5 (± 0.87)
**Week 1**	**Control**	10.0 (7.38-12.63)	---------	Not achieved
	**Pyrethroid**	1.0 (0.00-0.00)	36.6 (*P* < 0.001)	10.5 (± 2.02)
	***B. bassiana***	4.0 (3.76-4.25)	65.5 (*P* < 0.001)	5.8 (± 0.75)
**Month 1**	**Control**	9.0 (7.82-10.19)	---------	12.3 (± 0.48)
	**Pyrethroid**	1.0 (0.00-0.00)	107.9 (*P* < 0.001)	3.0 (± 1.22)
	***B. bassiana***	7.0 (6.05-7.59)	3.5 (*P* = 0.060)	11.5 (± 1.04)
**Month 2**	**Control**	11.0 (10.14-11.86)	---------	Not achieved
	**Pyrethroid**	1.0 (0.00-0.00)	88.19 (*P* < 0.001)	5.0 (± 2.31)
	***B. bassiana***	7.0 (6.46-7.54)	80.4 (*P* < 0.001)	9.3 (± 0.75)
**Month 3**	**Control**	10.0 (9.07-10.93)	---------	Not achieved
	**Pyrethroid**	9.0 (8.25-9.75)	5.7 (*P* = 0.017)	7.5 (± 3.75)
	***B. bassiana***	4.0 (3.74-4.26)	168.7 (*P* < 0.001)	Not achieved
**Month 4**	**Control**	Not achieved	---------	Not achieved
	**Pyrethroid**	Not achieved	41.9 (*P* < 0.001)	Not achieved
	***B. bassiana***	Not achieved	11.3 (*P* = 0.001)	Not achieved
**Month 5**	**Control**	Not achieved	---------	Not achieved
	**Pyrethroid**	2.0 (1.69-2.31)	122.9 (*P* < 0.001)	Not achieved
	***B. bassiana***	Not achieved	0.92 (*P* = 0.34)	Not achieved

‘Not achieved’ indicates that the desired level of mortality (50% - Median Lethal Time or 80%) was not reached before the end of the 14 day monitoring period.

Overall there was quite large and unexpected variation in control survival between months. This sometimes obscured treatment effects. For example, in month 1 on concrete there was no difference between fungus and controls because control mortality was high. There was a clear effect of fungus in subsequent months as control survivorship improved.

#### Assay 2 – *An. gambiae*

**Figure 5:**
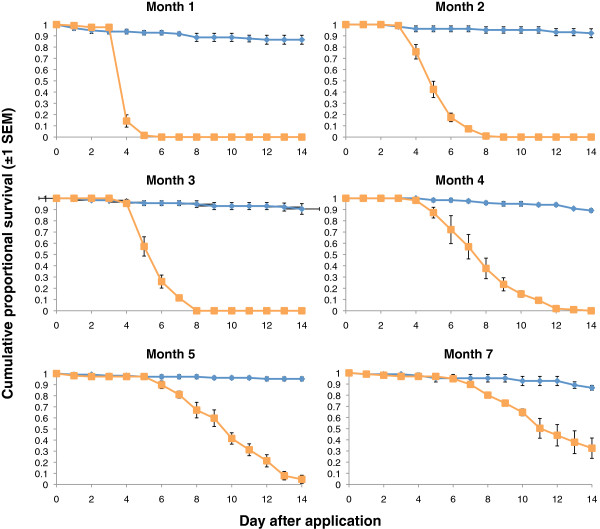
**Cumulative proportional survival of *Anopheles gambiae *exposed to spores of *B. bassiana *.** Control tiles were left untreated. Exposures were carried out monthly after an initial post application assessment (Day 1 – not shown, see Table 5) for seven months. Blue and orange curves show survival for the control and *B. bassiana* treatments respectively.

Exposure to spores on the clay substrate produced >80% mortality up to month 5. Unfortunately an accident in the lab compromized the collection of the month 6 data. At month 7 the day 14 mortality was still considerable but had dropped to around 68% (Table 5 Figure 5).

**Table 5:** ***Anopheles gambiae *survival after to clay tiles**

**Time after exposure**	**Treatment**	**Median Lethal Time in days (95% C.I.)**	**Log rank statistic (Significance compared to control)**	**Time to 80% mortality days (± 1 SEM)**
**Day 1**	**Control**	Not achieved	---------	Not achieved
	***B. bassiana***	5.0 (4.81-5.19)	209.0 (*P* < 0.001)	5.8 (± 0.48)
**Month 1**	**Control**	Not achieved	---------	Not achieved
	***B. bassiana***	4.0 (3.85-4.15)	185.9 (*P* < 0.001)	4.5 (± 0.29)
**Month 2**	**Control**	Not achieved	---------	Not achieved
	***B. bassiana***	5.0 (4.72-5.28)	208.1 (*P* < 0.001)	6.3 (± 0.25)
**Month 3**	**Control**	Not achieved	---------	Not achieved
	***B. bassiana***	6.0 (5.71-6.29)	219.6 (*P* < 0.001)	7.0 (± 0.00)
**Month 4**	**Control**	Not achieved	---------	Not achieved
	***B. bassiana***	8.0 (7.53-8.47)	236.9 (*P* < 0.001)	9.5 (± 0.65)
**Month 5**	**Control**	Not achieved	---------	Not achieved
	***B. bassiana***	10.0 (9.48-10.52)	155.4 (*P* < 0.001)	9.5 (± 0.65)
**Month 6**	**No assessments made**			
**Month 7**	**Control**	Not achieved	---------	Not achieved
	***B. bassiana***	11.0 (10.04-11.96)	55.5 (*P* < 0.001)	Not achieved

‘Not achieved’ indicates that the desired level of mortality (Median Lethal Time or 80%) was not reached before the end of the 14 day monitoring period.

On the cement and wood substrates, fungal exposed insects demonstrated significantly higher mortality than controls up to month 3 but never achieved the 80% mortality target (Tables 6 and 7). Accordingly, exposures were not conducted beyond month 3 for these treatments.

**Table 6:** ***Anopheles gambiae *survival after exposure to cement tiles**

**Time after application**	**Dose and Formulation**	**Median Lethal Time in days (95% C.I.)**	**Log rank statistic (Significance compared to control)**	**Time to 80% mortality days (± 1 SEM)**
**Day 1**	**Control**	Not achieved	---------	Not achieved
	***B. bassiana***	12.0 (10.28-13.72)	28.1 (*P* < 0.001)	Not achieved
**Month 1**	**Control**	Not achieved	---------	Not achieved
	***B. bassiana***	Not achieved	n.s.	Not achieved
**Month 2**	**Control**	Not achieved	---------	Not achieved
	***B. bassiana***	Not achieved	12.6 (*P* < 0.001)	Not achieved
**Month 3**	**Control**	Not achieved	---------	Not achieved
	***B. bassiana***	Not achieved	19.0 (*P* < 0.001)	Not achieved

‘Not achieved’ indicates that the desired level of mortality (50% - Median Lethal Time or 80%) was not reached before the end of the 14 day monitoring period.

**Table 7:** **Time of exposure, formulation and dose details and *Anopheles gambiae *survival estimates following exposure to *Beauveria bassiana *spores applied to a wood substrate**

**Time after application**	**Dose and Formulation**	**Median Lethal Time in days (95% C.I.)**	**Log rank statistic (Significance compared to control)**	**Time to 80% mortality days (± 1 SEM)**
**Day 1**	**Control**	Not achieved	---------	Not achieved
	***B. bassiana***	Not achieved	9.1 (*P* < 0.003)	Not achieved
**Month 1**	**Control**	Not achieved	---------	Not achieved
	***B. bassiana***	Not achieved	4.9 (*P* = 0.027)	Not achieved
**Month 2**	**Control**	Not achieved	---------	Not achieved
	***B. bassiana***	Not achieved	19.1 (*P* < 0.001)	Not achieved
**Month 3**	**Control**	Not achieved	---------	Not achieved
	***B. bassiana***	Not achieved	n.s.	Not achieved

‘Not achieved’ indicates that the desired level of mortality (50% - Median Lethal Time, or 80%) was not reached before the end of the 14 day monitoring period.

## Discussion

Spores of *Beauveria bassiana* stored under ideal conditions of low moisture content and low temperature survived with no loss of viability for over two years. Assuming access to cold storage, this baseline viability meets the WHO pesticide shelf-life criterion [27]. Once removed from refrigeration spore viability declined in a temperature dependent manner with high temperatures resulting in a faster rate of mortality than cooler temperatures. Surprisingly, under open storage, where the spore powder was exposed to high humidity in the incubators, survival was better than when spores were protected from humidity. Why this was so is unclear as maintaining low moisture content should improve spore stability [32]. Nonetheless, these results suggest the need for relatively rapid distribution and use of product once taken out of some sort of long-term stockpile. Such an approach is similar to standard practice for the mosquito larval biocontrol agent, *Bacillus thuringiensis*[45-47]. Moreover, certain commercial preparations of *B. bassiana* used in agriculture exhibit shelf lives of 12 months or more at room temperature [33,34,48], so there is scope for more product development. Further, data from other storage studies suggest that the viability of spores under variable temperature conditions (i.e. not refrigeration) might depend on the absolute age of the spores [44]. The current experiments used spores that had already been in storage for 585 days. Taking spores from storage at perhaps three or six months after production could potentially alter viability/persistence downstream. Assuming there is some sort of trade-off, optimizing time spent in storage *vs* time available for distribution and use will be an important factor in supply chain management.

Spore persistence following application varied considerably between substrates and assays. With the *An. stephensi* assays the control mortality was higher and much more variable than expected, making interpretation of results slightly difficult on some occasions. However, effective persistence was clearly demonstrated up to 4 months on clay substrates (and this could be extended to five months if mosquitoes contacted the treated substrates more than once). Persistence on cement and wood were less good, but 80% morality was still achieved up to months 2 and 3, respectively. Persistence of 2–4 months is within the range of existing chemical insecticides approved for use in IRS by the WHO [36,37]. Indeed, the fungus performed considerably better on the clay and concrete substrates than a basic formulation of Lambda-cyhalothrin, which essentially had no impact from month 1 (or even day 1 on clay). Performance of the chemical was much better on wood. These results are consistent with previous observations that porous substrates such as clay can interfere with the pick-up of chemical insecticides [37,49,50].

Control survival in the *An. gambiae* assays was much more stable, leaving treatment effects unambiguous. On clay tiles the fungal spray residue remained effective for between 5–7 months (the pattern of the mortality data suggest the month 6 exposure would have achieved the 80% cut off, but unfortunately the month 6 samples were not available). On wood and cement tiles, on the other hand, while there was some significant mortality relative to controls for up to 3 months, mortality of 80% was never achieved even from day 1.

The reasons for the differences in persistence and efficacy of fungal spray residues between substrates are unclear and we have no satisfactory explanation as to why spray applications on both wood and cement produced such different results between assays. Physical removal of spores from the substrates by mosquitoes could have occurred but did not obviously contribute to the patterns of decline we saw (i.e. the substrates that remained viable longest actually had the greatest number of repeat exposures). Cement is strongly alkaline and it is possible that this impacted spore survival relative to clay. High pH has been shown to affect persistence of chemical active ingredients [50]. The poorer effective persistence on wood was slightly more surprising as we had expected it to be a relatively inert, non-absorptive substrate. A previous study examining long-term survival of spores of the current fungal isolate sprayed on glass slides (also inert and non porous), reported a half-life >3 months [51]. It is possible, therefore, that the commercial plywood we used had some sort of chemical treatment that affected spore viability. Our attempts to verify with the seller that the plywood was completely free of antifungal chemicals were unsuccessful. There are no recommendations in the WHOPES guidelines for exactly what sort of substrates to test beyond wood, clay and cement. Further studies examining different types of these basic substrates would be worthwhile.

Overall, the results of the current study are extremely encouraging. Fungal spores can be produced and stored without loss of viability for >2 years. The fungus is a living organism and once removed from storage, spores are relatively sensitive to temperature but there appears scope for further product development work to improve stability, as well as options for appropriate supply chain management similar to that used for other biologicals. Once sprayed, simple oil formulations can persist as long as some of the standard chemical insecticides. Performance on clay was especially good, which is encouraging as the porous properties of clay/mud are challenging for chemical insecticides. Performance on the other substrates was more variable, but no more so than the basic wettable powder formulation of Lambda-cyhalothrin, which is one of the most widely used chemicals in IRS [52,53]). More advanced formulations of Lambda-cyhalothrin, such as capsulated suspensions, have been developed and shown to enhance performance in the field [52,53]. It is highly likely that with equivalent research effort, novel formulations of fungal pathogens (including microencapsulation [54-56]) could also be developed to further improve shelf life and persistence. Furthermore, in line with WHO protocols, assessments in the current study focused on mortality effects alone. While conventional chemicals based on fast-acting neurotoxins do result in rapid death, slower speed of kill (i.e. high mortality by day 14 when mosquitoes could conceivably transmit malaria) is sufficient to provide good malaria control assuming good product coverage see [24-26]. Moreover, it has been demonstrated that sub- or pre-lethal effects of fungal infection can add to mortality to reduce transmission potential further [6,7,23]. As such, it is probable that levels of persistence reported here are underestimates of overall effective persistence.

## Competing interests

The authors declare that they have no competing interests.

## Authors’ contributions

SB, AFR and MBT conceived and designed the experiments. SB, BHKC, RC, OM and NL conducted the experiments. NEJ, LK, MC provided fungal and entomological materials and laboratory resources. SB analysed the results. SB, AFR and MBT drafted the manuscript. All authors read and approved the final manuscript.
